# Emerging engineering principles for yield improvement in microbial cell design

**DOI:** 10.5936/csbj.201210016

**Published:** 2012-12-09

**Authors:** Santiago Comba, Ana Arabolaza, Hugo Gramajo

**Affiliations:** a Microbiology Division, IBR (Instituto de Biología Molecular y Celular de Rosario), Consejo Nacional de Investigaciones Científicas y Técnicas, Facultad de Ciencias Bioquímicas y Farmacéuticas, Universidad Nacional de Rosario, Suipacha 531, (S2002LRK) Rosario, Argentina

## Abstract

Metabolic Engineering has undertaken a rapid transformation in the last ten years making real progress towards the production of a wide range of molecules and fine chemicals using a designed cellular host. However, the maximization of product yields through pathway optimization is a constant and central challenge of this field. Traditional methods used to improve the production of target compounds from engineered biosynthetic pathways in non-native hosts include: codon usage optimization, elimination of the accumulation of toxic intermediates or byproducts, enhanced production of rate-limiting enzymes, selection of appropriate promoter and ribosome binding sites, application of directed evolution of enzymes, and chassis re-circuit. Overall, these approaches tend to be specific for each engineering project rather than a systematic practice based on a more generalizable strategy. In this mini-review, we highlight some novel and extensive approaches and tools intended to address the improvement of a target product formation, founded in sophisticated principles such as dynamic control, pathway genes modularization, and flux modeling.

## Introduction

The utilization of industrial biotechnology for the conversion of energy, chemicals and materials to value-added products is centuries-old. Traditional methods of strain development based on evolution, random mutagenesis, mating and selection strategies were successfully used for the optimization of production hosts. However, early applications of microbial production of chemicals were limited to native metabolites, such as amino acids, alcohols, organic acids, fatty acids or antibiotics. The foundational development of genetic and metabolic engineering made possible the production of a wide and diverse range of molecules including biofuels, pharmaceuticals, biopolymers, precursors and specialty chemicals [1]. By definition, metabolic engineering is the field that involves the construction, redirection, and manipulation of cellular metabolism through the alteration of endogenous and/or heterologous enzyme activities and levels to achieve the biosynthesis or biocatalysis of desired molecules [2]. Thus, the design of a cell factory requires a thoughtful understanding of the metabolic reactions involved in the synthesis of a target product, the consideration of the regulatory elements that affect metabolic throughput and the analysis of interconnectedness of cellular metabolism. In this sense, the development of the collectively named “omics technologies” has prompted the progress of metabolic engineering in the past decades: 1) the improvements in genome sequencing and DNA synthesis technologies; 2) the expansion of gene expression, metabolic reactions and enzyme structures databases; 3) the generation of new genetic tools to exert a strict control over metabolic pathways; 4) the setting up of new analytical methods to detect and quantify RNA, protein and cell metabolites; and 5) the creation of detailed biological models aided to the design of enzymes and metabolic pathways [3].

The potential of metabolic engineering could be exemplified by the early successful heterologous production of polyketide compounds. Polyketides are an important class of natural products with complex chemical structures widely used as antibiotics, immunosuppressant, antitumor, antifungal and antiparasitic agents [4]. These biomolecules are synthesized from simple building blocks such as acetyl coenzyme A (acetyl-CoA), propionyl-CoA, malonyl-CoA, and methylmalonyl-CoA through the action of multienzyme complexes or large modular megasynthases called PKSs [5]. The structural complexity of polyketides often precludes the development of practical chemical synthetic routes, leaving fermentation as the only viable source for the commercial production of these pharmaceutically and agriculturally useful agents. Since most polyketide-producing organisms are often difficult to culture at industrial scale, and the genetic tools available for them are scarce, the use of a more genetically and physiologically tractable heterologous host became a reasonable alternative. A significant achievement in this field was the production of the 6-deoxyerythronolide B (6dEB) aglycone in an engineered strain of *Escherichia coli* [6]. This was followed by the successful biosynthesis of yersiniabactin [7], a polyketide-nonribosomal peptide hybrid, and an ansamycin polyketide precursor [8]. These results provide the platform, not only for the expression of new clusters of PKS genes but also to greatly enhance the rate at which combinatorial biosynthesis tools are developed and PKS are engineered. Remarkably, the expression of 23 foreign genes in *E. coli* enabled the conversion of 6dEB into the bioactive erythromycin analogs Ery C and Ery D. Further chassis optimization provided a robust proof of principle that mature erythromycin analogs can be efficiently produced in a single heterologous expression system [9, 10].

Nevertheless, metabolic engineering is not only restricted to “compound manufacture”. Nielsen J. grouped different examples of metabolic engineering into the following categories [11]: 1) heterologous protein production; 2) extension of substrate range; 3) creation of pathways leading to new products; 4) degradation of xenobiotics; 5) engineering of cellular physiology for process improvement; 6) elimination or reduction of byproduct formation; 7) improvement of yield and productivity.

Maximization of product yields through pathway optimization is a central challenge for every metabolic engineering project [3]. Natural metabolic pathways are controlled by myriad of regulatory systems such as global and specific transcription factors, promoter strength, biochemical regulation of enzymes and substrate availability, among others. Metabolic engineers can potentially repurpose these features to modulate pathway components in order to improve the efficiency of product formation. In this sense, the ability to tune pathways has improved as the fundamental principles of metabolism and biological regulation continue to be discovered [12]. Because the yield and productivity of a process are linked to its commercial viability, the optimization of production of a target metabolite often requires precisely controlled expression of several natural and/or heterologous genes to avoid that individual conversion steps in the pathway limit the desired product yield, ensuring that cellular resources and energy are being efficiently utilized.

In essence, pathway optimization is a multivariate problem and the answer of this challenge is not universal and depends on the pathway to be expressed, the compound to be produced, the host, the availability of genetic tools, and the substrates utilized. In this context, the ability of the metabolic engineer to identify potential bottlenecks and eliminate them is critical and has a decisive impact on project success. This mini-review will dig into the existing novel approaches intended to improve the efficiency of metabolite-compounds production in designed microbial cells.

## Dynamic control of biosynthetic pathways to balance metabolism

The simple overexpression of a biosynthetic pathway often is not enough to achieve high yield production of the desired compound. In this context, the depletion of essential cellular precursors (for the production of needless RNAs, proteins or metabolites) and the accumulation of intermediates can cause toxic effects on the host and lead therefore to a decrease in productivity [13–15]. On the other hand, the suboptimal expression of the genes coding the rate-limiting enzymes of a metabolic pathway will also limit the production of the target compound. Metabolic flux imbalances are always governing the yield of a desired product, therefore methods to tightly control gene expression and/or protein levels are essential in the design of any metabolic engineering approach. Besides well known “static” methods or devices used to regulate gene transcription and RNA translation, such as modification of promoter strength, customization of RBS and modulation of RNA stability [16]; novel and more flexible strategies are being designed to exert a dynamic control over the heterologous systems. For example, dynamic regulation would allow an organism to adapt its metabolic flux to changes within the host or in its environment in real time [17]. An even better regulation system for an engineered pathway would sense the concentration of critical pathway intermediates and dynamically regulate the production and consumption of the intermediates, which would allow the delivery of intermediates at the appropriate levels and rates, in order to optimize the pathway for its highest productivity as conditions change in the cell's environment [18]. The common principle of these novel strategies is the application of “sensor-actuator systems” based either in proteins (specifically transcription factors) or in RNA-regulatory elements that could exert a fine and tunable control over the level of gene expression in a concerted manner by sensing the intracellular pool of a specific metabolite.

**Figure 1:**
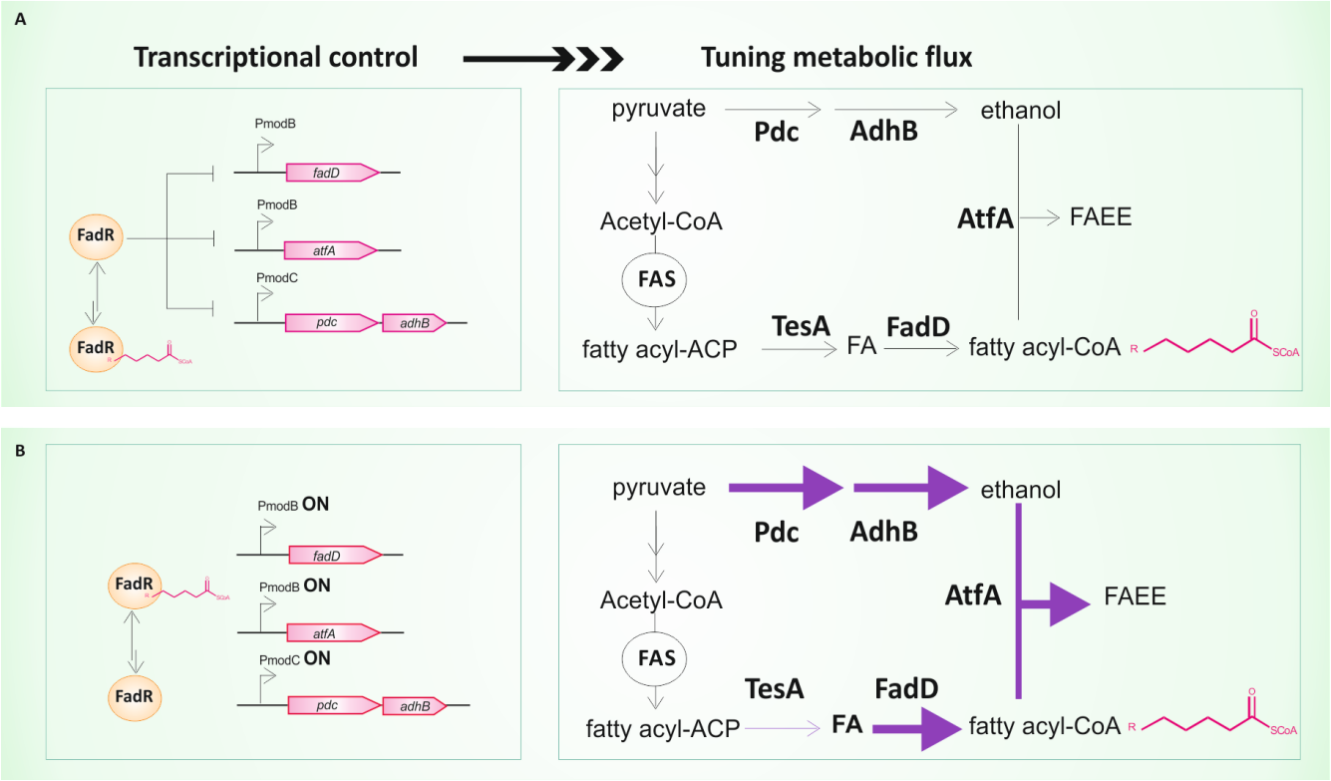
Biosensor-derived promoters upstream of modules B and C to control the expression of *fadD* and *atfA*, and the ethanol biosynthetic pathway (*adhB* and *pdc*), respectively. (A) FadR represses production of ethanol and unnecessary fatty acyl-CoA when the fatty acids (FA) concentration is low. (B) When the intracellular fatty acid concentration is sufficient, fatty acids would be first activated to fatty acyl-CoA (by chromosomal *fadD*) and then fatty acyl-CoA would release FadR from its DNA-binding sites. This would result in the induction of genes that encode enzymes to produce ethanol and *fadD* to generate more fatty acyl-CoA, and AtfA to convert ethanol and fatty acyl-CoA to FAEE. Increasing enzyme flux in response to fatty acyl-CoA is represented by the thickness of the arrows. FAS: Fatty Acid Synthase

Farmer and Liao introduced this idea by demonstrating that dynamic control of a flux could improve yields and productivity of lycopene production with an engineered *E. coli* strain [19]. The system was design to sense an excess of glucose by using an acetyl phosphate-activated transcription factor and its target promoter. Excess glucose flux and diversion of carbon to acetate formation increased the intracellular concentration of acetyl phosphate and reduced the productivity of lycopene. Thus, when acetyl phosphate accumulates inside the cell the transcription of two genes of the engineered pathway was up-regulated in order to redirect carbon flux from acetate to lycopene. This engineered strain produced 180-fold higher titers of lycopene than the strain that contains the biosynthetic genes under control of *tac* promoter [19]. Recently, Zhang et al. described a dynamic sensor-regulator system (DSRS) to optimize a fatty acid-derivative compound production in *E. coli* [18]. DSRS is based on a ligand-responsive transcription factor that regulates the expression levels of genes involved in fatty acid ethyl ester (FAEE) production. In this system, a biosensor was design in order to coordinately regulate the expression of the enzymatic steps that provide ethanol and condense it with fatty acyl-CoA according to the availability of fatty acids in the cell (Figure 1). This biosensor is based on the *E. coli* FadR transcription factor, capable of binding fatty acyl-CoA molecules, and on hybrid promoters created by the combination of FadR and LacI responsive elements [18]. The FAEE biosynthetic pathway contains three modules. Module A uses the native *E. coli* fatty acid biosynthetic pathway and a thioesterase, TesA, to produce free fatty acids. Module B is an ethanol biosynthetic pathway that converts pyruvate into ethanol. Module C contains an acyl-CoA synthase (FadD) and a wax-ester synthase (AtfA). AtfA condenses the fatty acyl-CoA product (generated from module A plus FadD activity) and ethanol (module B) to form FAEEs (Figure 1). Following this approach, 20 different strains were constructed and the best ones reached a productivity of 1.5 g/l after 3 days of incubation, corresponding to 28% of the maximum FAEE theoretical yield [18].

Up to date, there are few published examples of engineered dynamic control of fluxes in heterologous pathways. However, in general, this strategy can be extended to design biosensors and regulatory systems for other molecules and metabolic pathways using the large pool of natural ligand-responsive transcription factors [20]. These systems should allow to sense in real time the metabolism of the host cells and adjust the expression of the biosynthetic machinery in such a way to prevent toxicity and maximize the efficiency of product formation.

The above mentioned mechanisms were based on proteins which are involved in regulating gene expression at the transcriptional level. However, as it is widely known, the modulation of gene expression can be achieved by intervening at post transcriptional stages. One remarkable example of a biological mechanism that has been successfully adapted to control the expression of engineered systems is based on the use of RNA-regulatory elements called riboswitches. These natural elements can recognize a variety of structurally diverse ligands, controlling gene expression in a concentration dependent manner [21]. Moreover, RNA elements can be subjected to successive rounds of mutagenesis and selection in order to generate synthetic riboswitches that could respond to a desired compound. Building on natural examples, a variety of synthetic RNA regulators have been designed to control gene expression. Some interesting examples are: 1) riboswitches that control bacterial gene expression in a theophylline-dependent fashion [22]; 2) RNA switches (termed riboregulators) that have been developed in *E. coli* to activate translation in response to RNA signals [23]; 3) an atrazine-responsive RNA switch coupled to *cheZ* gene to control bacterial cell motility, allowing cells to move along a gradient of the pollutant [24]. A clear progress in RNA-based metabolic engineering endeavors resulted in the development of a modular platform for constructing molecular sensors to noninvasively detect biosynthesis of a target metabolite in real-time through a fluorescent reporter signal. The successful application of these switches was demonstrated in the detection of xanthine when a precursor was fed to the cell cultures [25]. Future advances in the design of synthetic RNA switches may extend their application in metabolic engineering to exert spatial, temporal and dynamical control within biosynthetic pathways.

## Modularization and systematization of metabolic engineering

As we already mentioned, classical optimization of metabolic pathways is achieved by modification of promoters, RBS, codon composition, copy number, construction of operons, etc [26]. However, these approaches can rarely be extrapolated among different hosts since the genetic toolboxes available for each microorganism differ greatly and the understanding of regulatory mechanisms prevailing into the defined cell host is often incomplete. This scenario thus implies that an almost complete optimizing strategy has to be designed *ad hoc* for each new metabolic engineering project, making this discipline costly and very time-consuming [27].

In order to achieve the same flexibility of chemical engineering for the production of structurally diverse molecules, metabolic engineering need to establish principles of systematization to achieve their goals in terms of production and optimization. An excellent application of this concept was applied to the design and development of engineered pathways for the production of terpenoid derived moieties.

Studies addressing the heterologous pathway optimization of isoprenoid-derived molecules on *E. coli* resulted in innovative and interesting advances not only for their particular yield improvement, but also because they laid down strategies capable to systematize metabolic engineering approaches, generating universal principles capable of been transferred to other pathways and/or hosts [27]. Chronologically, this case moved from particular to more universal optimization principles developed and applied in order to maximize product titers of these pathways. Due to their polymer-forming functional groups, the natural terpenoid family of products is composed of very diverse functional and structural molecules, ranging from antioxidant pigments to therapeutic drugs. Amorphadiene (amorpha-4,11-diene) and taxadiene (taxa-4,11-diene) are precursors for the synthesis of the antimalarial compound artemisinin and the anticancer drug taxol, respectively. In nature, terpenoids are synthesized by one of two possible routes: the mevalonate (MVA) pathway, which starts with the condensation of two acetyl-CoA and is present in eukaryotic cells, and the non-mevalonate 2-methyl-(D)-erythriol-4-phosphate (MEP) pathway, which initiates with the condensation of glyceraldehyde-3-phosphate and pyruvate, and can be found in bacteria and plant plastids [27].

First, amorphadiene production was achieved by heterologous expression of the MVA pathway from yeast and a codon-optimized amorphadiene synthase gene from *Artemisia annua*, in *E. coli* [28]. Although this system could produce significant levels of the precursor moiety, it was far from the theoretical maximum yield. Further optimization of amorphadiene titers was done by Anthony et al. [26]. Importantly, in this study the authors gave the first outline for systematization of amorphadiene metabolic engineering. They constructed a plasmid in which combinations of various promoters and operon constructs could be easily substituted and tested. In this way they addressed the problems of having many plasmids for high level of gene expression in the same cell (i.e., the metabolic burden imposed to the cell because of the active synthesis of plasmid DNA, and the corresponding proteins for antibiotic resistance [29]; and the flux imbalances between the segments of the pathway which are in different replicons due to plasmid instability and fluctuations in copy numbers). Using this backbone they were able to combine on one vector two transcriptional units previously located in different plasmids, increasing the amorphadiene titers by 3- fold. Furthermore, they were able to identify the limiting enzymatic step by overexpressing one by one each gene of the pathway and quantifying the final amount of amorphadiene being produced. This approach constitutes a clear demonstration in which classical optimization techniques can be used in a systematic way, providing a strategy that can be transferred to other systems.

**Figure 2:**
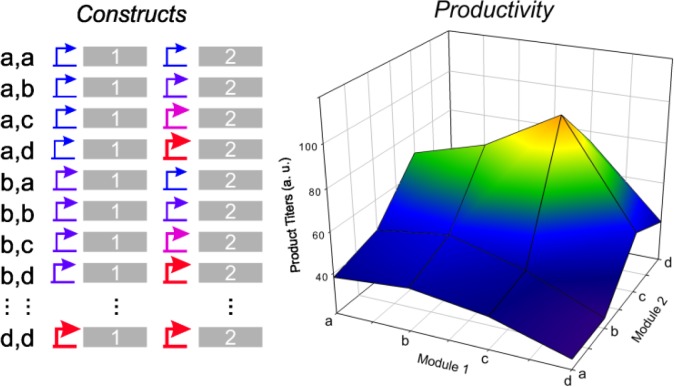
The genes of the biosynthetic pathway are grouped into modules 1 and 2 and different promoters are cloned upstream of each one. The generated strains contain all the possible combinations between the constructs. All these strains are then tested for the production of the target compound and the yield of product is plotted as a function of both modules.

Ajikumar et al., went further with systematization and they laid down a novel approach known as “multivariate modular metabolic modeling” (MMME) [30]. They developed a system for efficient taxadiene synthesis in *E. coli* by expressing the native host MEP pathway, the geranylgeranyl diphosphate (GGPP) synthase and the taxadiene synthase. The MEP pathway has the advantage over the MEV pathway in being more balanced and efficient in producing isoprenoids from sugars. The MMME approach is based on grouping enzymes with similar turnovers in modules, and then doing a combinatorial screening varying the expression of the different modules. For this, operons are constructed and the toolbox of promoters, RBS and plasmid vector can be used to achieve different expression levels which will maximize the product titers of the system. The combinatorial approach can easily circumvent the non-linear interactions between enzymes in the pathway and offer the opportunity to broadly sample parameter space [30]. In this way, the authors constructed the “upstream module”, an operon coding for the genes of the *E. coli* MPE pathway, which generates IPP (isopentenyl pyrophosphate) and DMAPP (dimethylallyl pyrophosphate); and the “downstream module”, a GGPP synthase and taxadiene synthase biscistronic transcriptional unit. Thus, instead of dealing with six genes, the problem is reduced to two modules, considerably simplifying the system, making it more tractable and easy to handle. In this way, the carbon flux balance along the pathway is achieved by changing the expression levels (plasmid copy number or promoter strength) of each module, and doing a combinatorial screening of all the possible combinations (Figure 2). Using this approach, the authors needed to construct 32 strains in order to obtain a substantial increase of 15,000 times in taxadiene titers over the control strain without modularization. In this case, this was enough to carefully balance the flux trough the heterologous pathway, avoiding the accumulation of toxic intermediaries and matching the output with the input of the upstream and downstream modules respectively. It is worth to mention that the same combinatorial approach without modularization involves the tuning of eight genes for MEP pathway and two more to reach taxadiene, implying the construction of at least 10,000 strains and the necessity of having a high-throughput screening method.

Despite its outstanding medical and industrial relevance, the progress in metabolic engineering of this family of products is laying down the basis for systematization of metabolic engineering in general. Principles of codon-adjustment, identification of pathway bottlenecks and, more recently, modularization and combinatorial experimentation can be transferred and reused to perform the heterologous production of completely different products. These principles, together with the ever declining costs of DNA synthesis and sequencing, and the development of computational software for analyzing and predicting the behavior of these heterologous systems will lead microbial metabolic engineering to be a systematic and flexible tool for production of an expanding array of molecules for pharmaceutical and other industrial uses.

## Metabolic network flux analysis for yield improvements

The last two decades encompassed what is called “post-genomics era” of metabolic engineering, an overwhelming compendium of data coming from genomics, transcriptomics, proteomics and more recently, metabolomics fields. In this context, the paradigm of metabolic engineering has clearly shifted away from one focused in perturbing individual pathways, to one which considers cell reactions in their entirety, and which integrates metabolic (native and heterologous) pathways in a genome-scale metabolic network.

Metabolic engineering makes particular emphasis in the metabolic fluxes and their control under *in vivo* conditions in order to maximize product formation [2]. Since empirical determination of the whole-cell metabolic fluxes is an almost impossible task, they have to be inferred from constraint based computational simulations that include measurable parameters (i.e. substrate uptake, growth rate) in their equations. Metabolic networks are described as a collection of the biochemical reactions that occur in the cell. Each of these reactions is linked to the plethora of putative enzymes found in the organism genome [31]. In the flux balance analysis (FBA) approaches, the set of reactions is displayed as a stoichiometric matrix, in which each reaction has an assigned constraint that defines the intervening molecules, the direction and the flow through it [32, 33]. Once defined the laying metabolic network, FBA seeks to maximize or minimize an objective function, which can be any linear combination of fluxes. Classically, the objective function for microorganisms is to maximize biomass-related reactions, i.e. simulating maximum growth. Thus by knowing a restricted set of measurable parameters, quantitative predictions and testable hypothesis can be generated [34]. By altering the constraints in the stoichiometric matrix, FBA can simulate different media or gene deletions, calculate the new metabolic flux distribution and predict the concomitant parameters of growth and yield [33, 35, 36].

While it is generally accepted that microorganisms metabolism is suited to achieve optimal growth, this may not be the case of a mutant organism obtained under laboratory conditions. Frequently, when a mutation is introduced, the topology and flux distribution of a metabolic network is so perturbed that the mutant organism does not follow the optimal growth principle. In such cases, the objective of “minimization of metabolic adjustment” (MOMA) is better suited for predicting growth and product yields, since it is not based on an optimal growth objective function, but in the concept that the mutant metabolism remains initially as close as possible to the wild type optimum in terms of flux values [34]. In this context, MOMA uses the same stoichiometric matrix as FBA, but the solution is a metabolic flux distribution which is an intermediate scenario between the optimal wild type and the optimal mutant flux distribution [34].

These two different approaches to analyze metabolic networks are frequently complementary, and understanding the general principles and the biological interpretation of each method provide an extremely powerful advantage to the metabolic engineer [31]. Algorithms such as COBRA 2.0 toolbox [37], OptKnock [38], OptFlux [39] and OptForce [40], among others, are available to suggest gene knockouts that *in silico* predict increasing product titers. For example, Brochado et al., have successfully improved vanillin production in yeast by knocking out genes of central carbon metabolism [41]. More recently, malonyl-CoA supply for heterologous production of target molecules was improved by means of genome-scale metabolic modeling analysis [40]. Malonyl-CoA is an essential building block for the production of many industrial and medical relevant polyketides [6, 42] and flavanones [43], and also the starting point for microdiesel [44]. Since malonyl-CoA is also the precursor for the essential fatty acid biosynthesis, the flux towards it is tightly controlled and the cell will not easily commit to overproduce recombinant molecules derived from such essential metabolites [45]. Many researchers have improved the availability of malonyl-CoA by tuning enzymatic steps that replenish or deplete malonyl-CoA pools [46]. However, Xu et al., provide the first rational approach of *in silico* analysis of carbon metabolism in *E. coli* for redirecting carbon flux to malonyl-CoA, in order to optimize the synthesis of the flavanone naringenin [40]. They used OptFORCE algorithm to identify the minimal set of genetic interventions that led to maximization of product titers. The main targets identified were: A) up-regulation of glycolytic enzymes; B) down-regulation of tricarboxylic acid flux; C) increased pyruvate dehydrogenase (PDH) and acetyl-CoA carboxylase (ACC) activities. In this way, the authors improved the production of naringenin by 560% over the current state of art by introducing a total of five genetic interventions: overexpression of ACC, PDH, glyceraldehyde-3-phosphate dehydrogenase and phosphoglycerate kinase, and a knockout of the succinyl-CoA synthetase and fumarase enzymes.

Competition with native pathways and metabolites, accumulation of side products and consequent growth inhibition are only some of the possible effects that a heterologous pathway can have on cell metabolism. These examples are a clear demonstration of how metabolic networks and metabolic flux analysis can be used to identify non-trivial targets for genetic interventions that lead to further optimization of product titers. *In silico* simulation tools open the field of metabolic engineering possibilities that can be explored to circumvent the problems that arise when expressing a foreign pathway, contributing to accelerate the design cycle of metabolic engineering and leading to the cost-effective production of industrial and medical important molecules.

## Perspectives

The rapid rate of fossil resources consumption has drawn the attention towards the development of an economy based on renewable biological raw materials. In this context, the term “Biorefineries” is used to describe biological entities capable of generating high value-aggregated directly from biomass, in an analogy with petroleum-based refineries [47]. Metabolic engineering thus appears as a key discipline, modifying and creating metabolic pathways in microbial hosts in order to produce complex chemicals of therapeutic and industrial relevance [26]. However, these pathways often alter significantly the hosts native metabolic network, by either increasing toxic metabolite levels or depleting essential building blocks for the cell, and thus resulting in sub-optimal yield of the desired product [12].

The intersection of metabolic engineering with other emerging areas of systems and synthetic biology presents exciting opportunities to develop solutions to many of the global challenges we face nowadays. In particular, these disciplines will be of fundamental importance to promote sustainable energy, reduce the environmental impact of several industrial processes and to improve the cell factory production of natural and semisynthetic drugs that are currently synthesized at very low yields, hopefully having an impact in the costs and availability of medicines.

## References

[1] Curran KA, Alper HS (2012) Expanding the chemical palate of cells by combining systems biology and metabolic engineering. Metab Eng 14: 289–297 22595280 10.1016/j.ymben.2012.04.006

[2] Stephanopoulos G (1999) Metabolic fluxes and metabolic engineering. Metab Eng 1: 1–11 10935750 10.1006/mben.1998.0101

[3] Keasling JD (2010) Manufacturing molecules through metabolic engineering. Science 330: 1355–1358 21127247 10.1126/science.1193990

[4] Staunton J, Weissman KJ (2001) Polyketide biosynthesis: a millennium review. Nat Prod Rep 18: 380–416 11548049 10.1039/a909079g

[5] Cane DE, Walsh CT, Khosla C (1998) Harnessing the biosynthetic code: combinations, permutations, and mutations. Science 282: 63–68 9756477 10.1126/science.282.5386.63

[6] Pfeifer BA, Admiraal SJ, Gramajo H, Cane DE, Khosla C (2001) Biosynthesis of complex polyketides in a metabolically engineered strain of *E. coli*. Science 291: 1790–1792 11230695 10.1126/science.1058092

[7] Pfeifer BA, Wang CC, Walsh CT, Khosla C (2003) Biosynthesis of Yersiniabactin, a complex polyketide-nonribosomal peptide, using *Escherichia coli* as a heterologous host. Appl Environ Microbiol 69: 6698–6702 14602630 10.1128/AEM.69.11.6698-6702.2003 PMC262314

[8] Watanabe K, Rude MA, Walsh CT, Khosla C (2003) Engineered biosynthesis of an ansamycin polyketide precursor in *Escherichia coli*. Proc Natl Acad Sci U S A 100: 9774–9778 12888623 10.1073/pnas.1632167100 PMC187841

[9] Peiru S, Menzella HG, Rodriguez E, Carney J, Gramajo H (2005) Production of the potent antibacterial polyketide erythromycin C in *Escherichia coli*. Appl Environ Microbiol 71: 2539–2547 15870344 10.1128/AEM.71.5.2539-2547.2005 PMC1087553

[10] Peiru S, Rodriguez E, Menzella HG, Carney JR, Gramajo H (2008) Metabolically engineered *Escherichia coli* for efficient production of glycosylated natural products. Microb Biotechnol 1: 476–486 21261868 10.1111/j.1751-7915.2008.00046.x PMC3815289

[11] Nielsen J (2001) Metabolic engineering. Appl Microbiol Biotechnol 55: 263–283 11341306 10.1007/s002530000511

[12] Boyle PM, Silver PA (2012) Parts plus pipes: synthetic biology approaches to metabolic engineering. Metab Eng 14: 223–232 22037345 10.1016/j.ymben.2011.10.003 PMC3293987

[13] Alper H, Fischer C, Nevoigt E, Stephanopoulos G (2005) Tuning genetic control through promoter engineering. Proc Natl Acad Sci U S A 102: 12678–12683 16123130 10.1073/pnas.0504604102 PMC1200280

[14] Jones KL, Kim SW, Keasling JD (2000) Low-copy plasmids can perform as well as or better than high-copy plasmids for metabolic engineering of bacteria. Metab Eng 2: 328–338 11120644 10.1006/mben.2000.0161

[15] Raab RM, Tyo K, Stephanopoulos G (2005) Metabolic engineering. Adv Biochem Eng Biotechnol 100: 1–17 16270654 10.1007/b136411

[16] Pfleger BF, Pitera DJ, Smolke CD, Keasling JD (2006) Combinatorial engineering of intergenic regions in operons tunes expression of multiple genes. Nat Biotechnol 24: 1027–1032 16845378 10.1038/nbt1226

[17] Holtz WJ, Keasling JD (2010) Engineering static and dynamic control of synthetic pathways. Cell 140: 19–23 20085699 10.1016/j.cell.2009.12.029

[18] Zhang F, Carothers JM, Keasling JD (2012) Design of a dynamic sensor-regulator system for production of chemicals and fuels derived from fatty acids. Nat Biotechnol 30: 354–359 22446695 10.1038/nbt.2149

[19] Farmer WR, Liao JC (2000) Improving lycopene production in *Escherichia coli* by engineering metabolic control. Nat Biotechnol 18: 533–537 10802621 10.1038/75398

[20] Wilkinson SP, Grove A (2006) Ligand-responsive transcriptional regulation by members of the MarR family of winged helix proteins. Curr Issues Mol Biol 8: 51–62 16450885

[21] Barrick JE, Breaker RR (2007) The distributions, mechanisms, and structures of metabolite-binding riboswitches. Genome Biol 8: R239 17997835 10.1186/gb-2007-8-11-r239 PMC2258182

[22] Suess B, Fink B, Berens C, Stentz R, Hillen W (2004) A theophylline responsive riboswitch based on helix slipping controls gene expression *in vivo*. Nucleic Acids Res 32: 1610–1614 15004248 10.1093/nar/gkh321 PMC390306

[23] Callura JM, Dwyer DJ, Isaacs FJ, Cantor CR, Collins JJ (2010) Tracking, tuning, and terminating microbial physiology using synthetic riboregulators. Proc Natl Acad Sci U S A 107: 15898–15903 20713708 10.1073/pnas.1009747107 PMC2936621

[24] Sinha J, Reyes SJ, Gallivan JP (2010) Reprogramming bacteria to seek and destroy an herbicide. Nat Chem Biol 6: 464–470 20453864 10.1038/nchembio.369 PMC2873063

[25] Win MN, Smolke CD (2007) A modular and extensible RNA-based gene-regulatory platform for engineering cellular function. Proc Natl Acad Sci U S A 104: 14283–14288 17709748 10.1073/pnas.0703961104 PMC1964840

[26] Anthony JR, Anthony LC, Nowroozi F, Kwon G, Newman JD, et al. (2009) Optimization of the mevalonate-based isoprenoid biosynthetic pathway in Escherichia coli for production of the anti-malarial drug precursor amorpha-4,11-diene. Metab Eng 11: 13–19 18775787 10.1016/j.ymben.2008.07.007

[27] Yadav VG, De Mey M, Lim CG, Ajikumar PK, Stephanopoulos G (2012) The future of metabolic engineering and synthetic biology: towards a systematic practice. Metab Eng 14: 233–241 22629571 10.1016/j.ymben.2012.02.001 PMC3615475

[28] Martin VJ, Pitera DJ, Withers ST, Newman JD, Keasling JD (2003) Engineering a mevalonate pathway in *Escherichia coli* for production of terpenoids. Nat Biotechnol 21: 796–802 12778056 10.1038/nbt833

[29] Rozkov A, Avignone-Rossa CA, Ertl PF, Jones P, O'Kennedy RD, et al. (2004) Characterization of the metabolic burden on *Escherichia coli* DH1 cells imposed by the presence of a plasmid containing a gene therapy sequence. Biotechnol Bioeng 88: 909–915 15532038 10.1002/bit.20327

[30] Ajikumar PK, Xiao WH, Tyo KE, Wang Y, Simeon F, et al. (2010) Isoprenoid pathway optimization for Taxol precursor overproduction in *Escherichia coli*. Science 330: 70–74 20929806 10.1126/science.1191652 PMC3034138

[31] Copeland WB, Bartley BA, Chandran D, Galdzicki M, Kim KH, et al. (2012) Computational tools for metabolic engineering. Metab Eng 14: 270–280 22629572 10.1016/j.ymben.2012.03.001 PMC3361690

[32] Varma A, Palsson BO (1994) Stoichiometric flux balance models quantitatively predict growth and metabolic by-product secretion in wild-type *Escherichia coli* W3110. Appl Environ Microbiol 60: 3724–3731 7986045 10.1128/aem.60.10.3724-3731.1994 PMC201879

[33] Orth JD, Thiele I, Palsson BO (2010) What is flux balance analysis?. Nat Biotechnol 28: 245–248 20212490 10.1038/nbt.1614 PMC3108565

[34] Segre D, Vitkup D, Church GM (2002) Analysis of optimality in natural and perturbed metabolic networks. Proc Natl Acad Sci U S A 99: 15112–15117 12415116 10.1073/pnas.232349399 PMC137552

[35] Edwards JS, Palsson BO (2000) Metabolic flux balance analysis and the *in silico* analysis of *Escherichia coli* K-12 gene deletions. BMCBioinformatics 1: 1 10.1186/1471-2105-1-1 PMC29061 11001586

[36] Edwards JS, Ibarra RU, Palsson BO (2001) *In silico* predictions of *Escherichia coli* metabolic capabilities are consistent with experimental data. Nat Biotechnol 19: 125–130 11175725 10.1038/84379

[37] Schellenberger J, Que R, Fleming RM, Thiele I, Orth JD, et al. (2011) Quantitative prediction of cellular metabolism with constraint-based models: the COBRA Toolbox v2.0. Nat Protoc 6: 1290–1307 21886097 10.1038/nprot.2011.308 PMC3319681

[38] Burgard AP, Pharkya P, Maranas CD (2003) Optknock: a bilevel programming framework for identifying gene knockout strategies for microbial strain optimization. Biotechnol Bioeng 84: 647–657 14595777 10.1002/bit.10803

[39] Rocha I, Maia P, Evangelista P, Vilaca P, Soares S, et al. (2010) OptFlux: an open-source software platform for *in silico* metabolic engineering. BMC Syst Biol 4: 45 20403172 10.1186/1752-0509-4-45 PMC2864236

[40] Xu P, Ranganathan S, Fowler ZL, Maranas CD, Koffas MA (2011) Genome-scale metabolic network modeling results in minimal interventions that cooperatively force carbon flux towards malonyl-CoA. Metab Eng 13: 578–587 21763447 10.1016/j.ymben.2011.06.008

[41] Brochado AR, Matos C, Moller BL, Hansen J, Mortensen UH, et al. (2010) Improved vanillin production in baker's yeast through *in silico* design. Microb Cell Fact 9: 84 21059201 10.1186/1475-2859-9-84 PMC2992047

[42] Yuzawa S, Kim W, Katz L, Keasling JD (2012) Heterologous production of polyketides by modular type I polyketide synthases in *Escherichia coli*, Curr Opin Biotechnol. 10.1016/j.copbio.2011.12.029 22244790

[43] Leonard E, Yan Y, Fowler ZL, Li Z, Lim CG, et al. (2008) Strain improvement of recombinant *Escherichia coli* for efficient production of plant flavonoids. Mol Pharm 5: 257–265 18333619 10.1021/mp7001472

[44] Kalscheuer R, Stolting T, Steinbuchel A (2006) Microdiesel: *Escherichia coli* engineered for fuel production. Microbiology 152: 2529–2536 16946248 10.1099/mic.0.29028-0

[45] Pitera DJ, Paddon CJ, Newman JD, Keasling JD (2007) Balancing a heterologous mevalonate pathway for improved isoprenoid production in *Escherichia coli*. Metab Eng 9: 193–207 17239639 10.1016/j.ymben.2006.11.002

[46] Lim CG, Fowler ZL, Hueller T, Schaffer S, Koffas MA (2011) High-yield resveratrol production in engineered *Escherichia coli*. Appl Environ Microbiol 77: 3451–3460 21441338 10.1128/AEM.02186-10 PMC3126431

[47] Kamm B, Kamm M (2004) Principles of biorefineries. Appl Microbiol Biotechnol 64: 137–145 14749903 10.1007/s00253-003-1537-7

